# Deep plowing improves soil physical structure and alters nitrogen-cycling microbial communities in a subtropical red soil region

**DOI:** 10.3389/fmicb.2025.1734649

**Published:** 2026-01-12

**Authors:** Xiangyun Li, Yinuo Liu, Yongyu Pu, Guangcai Shen, Siman Gao, Shuai Kuang, Wenjing Song, Ping Cong

**Affiliations:** 1 Tobacco Research Institute of Chinese Academy of Agricultural Sciences, Qingdao, China; 2 Baoshan Branch of Yunnan Tobacco Company, Baoshan, China; 3 College of Resources and Environment, Shandong Agricultural University, Taian, China

**Keywords:** co-occurrence network, nitrogen cycling, soil enzyme activity, soil microbial community, tillage intensity

## Abstract

**Introduction:**

Tillage depth plays a critical role in regulating soil structure, nutrient dynamics, and microbial processes that determine crop productivity and quality.

**Methods:**

A 3-year field experiment was conducted in a subtropical red soil region to evaluate the effects of rotary tillage (RT), deep tillage with middle depth (DTM), and deep plowing (DP) on soil physicochemical properties, enzyme activities, microbial biomass, and nitrogen-cycling microbial communities.

**Results and discussion:**

Results showed that RT significantly enhanced soil organic carbon, total nitrogen, available phosphorus, and microbial biomass across both 0–20 cm and 20–40 cm layers. In contrast, DP promoted higher nitrate levels, particularly in subsoils, and significantly enhanced ammonia-oxidizing bacteria (AOB) diversity in the tillage layer. Soil enzyme activities showed depth-dependent responses, with RT maintaining greater cellulase and urease activity compared to other treatments. Ammonia-oxidizing archaea (AOA) and AOB communities exhibited distinct compositional and diversity shifts under different tillage regimes, driven by soil nutrient availability, bulk density, and enzymatic activity. Co-occurrence network analysis further revealed that DTM and DP increased the complexity of AOA networks, while RT promoted higher connectivity and modularity in AOB networks. Additionally, DP significantly increased the proportion of superior-grade leaves compared to RT. Path analysis further clarified soil microbial biomass as the strongest positive direct driver of production benefits, mediating the effects of soil physicochemical and nutrient properties on crop value. These findings indicate that deep plowing boosts agricultural economic returns by optimizing the soil environment to enhance crop quality; however, to offset accelerated organic matter decomposition, integrating deep plowing with organic amendments is recommended for sustainable production.

## 1 Introduction

Tillage practices play a pivotal role in shaping soil ecosystems through mechanical disturbance (Liu et al., 2024; Nunes et al., 2020; Roger-Estrade et al., 2010). Rotary tillage (RT), which breaks up the root layer and incorporates crop residues into the topsoil, can enhance surface nutrient availability and soil porosity (Shen et al., 2024). However, continuous RT may lead to soil compaction, reduced tillage depth, and hindered root penetration (Hamza and Anderson, 2005; Liu et al., 2010). In contrast, deep plowing (DP) involves turning over soil layers below 30 cm, thereby expanding the tillage layer, improving subsoil accessibility, and promoting deeper root growth (Feng et al., 2020; Schneider et al., 2017). Despite its agronomic benefits, the large-scale adoption of continuous deep tillage has historically been limited due to its high labor and energy demands (Damanauskas and Janulevičius, 2022). With advancements in agricultural machinery, the operational costs of deep tillage have declined, making it a more feasible practice (Goyal et al., 2014). While the adverse impacts of rotary or shallow tillage on surface soil properties are well documented, the effects of deep tillage on soil nutrient dynamics, microbial communities and their subsequent influence on crop yield and quality remain insufficiently understood, highlighting an urgent need for systematic investigation.

Soil microorganisms are key mediators of nutrient transformations, and tillage-induced alterations in soil physicochemical conditions inevitably affect microbial diversity and ecological function (Dhaliwal et al., 2024; Hartmann and Six, 2023). Among these microorganisms, ammonia-oxidizing archaea (AOA) and ammonia-oxidizing bacteria (AOB) are essential drivers of nitrification, catalyzing the conversion of ammonium (NH_4_^+^) to nitrate (NO_3_^−^) (Konneke et al., 2005; Venter et al., 2004). This process directly regulates nitrogen (N) availability for plant uptake and significantly influences N losses via leaching and the emission of nitrous oxide (N_2_O)—a potent greenhouse gas with a global warming potential approximately 298 times that of CO_2_ (Goyal and Qanungo, 2023; Ravishankara et al., 2009). Therefore, under current global climate mitigation strategies and China’s “dual carbon” goals, investigating how different tillage depths affect the community of AOA and AOB is crucial for improving N management, enhancing crop performance and reducing environmental risks in agroecosystems.

Studies have shown that AOA tend to dominate in environments with low ammonium availability, high acidity, or drought, while AOB are more prevalent under conditions of high ammonium concentration and good aeration (Di et al., 2009; Kozlowski et al., 2016; Li Q. et al., 2024). Given these distinct ecological niches, tillage practices play a pivotal role in shaping their community structure by altering the soil environment (Hartmann and Six, 2023). Different tillage intensities significantly alter the physical protection and vertical distribution of soil organic matter, which directly regulates nitrogen mineralization rates and substrate availability (Kristensen et al., 2003; Wang et al., 2023). For instance, intensive tillage disrupts soil aggregates and enhances aeration, accelerating the mineralization of organic nitrogen into ammonium, which typically favors AOB (Prosser and Nicol, 2012). In contrast, reduced tillage or deep plowing may create distinct gradients of organic matter and oxygen, potentially forming niches that support AOA or specific AOB taxa depending on the depth and intensity of disturbance (Prosser and Nicol, 2012). Consequently, variations in soil porosity, oxygen availability, and N forms induced by different tillage regimes drive shifts in the relative dominance and activity of these two groups (Li et al., 2015; Zhou et al., 2021). Therefore, understanding how tillage depth modulates the activity patterns of AOA and AOB remains crucial for elucidating the underlying microbial mechanisms driving N transformations.

In addition, microbial co-occurrence network analysis has recently gained increasing attention in soil ecology as a powerful tool to uncover potential cooperative or antagonistic interactions among microbial taxa (Barberán et al., 2012; de Vries et al., 2018). These analyses provide new insights into ecosystem stability, functional redundancy, and microbial resilience under environmental perturbations (Hernandez et al., 2021; Shi et al., 2020; Xue et al., 2022). Previous studies have demonstrated that changes in land-use patterns and tillage practices can significantly alter the topological structure of soil microbial networks, thereby affecting their resistance to disturbance and ecological functions (Li et al., 2022; Xue et al., 2022). However, studies focusing specifically on N-cycling microbes—particularly AOA and AOB—and their co-occurrence patterns under different tillage depths remain limited. Therefore, constructing co-occurrence networks of ammonia-oxidizing microorganisms and exploring their responses to variations in tillage depth may help to reveal novel regulatory mechanisms of soil N cycling.

In this study, a three-year field experiment was conducted in a tobacco cultivation system in a subtropical red soil region, where three tillage depths were tested: rotary tillage (RT), deep tillage with middle depth (DTM), and deep plowing (DP). We systematically evaluated the effects of these tillage treatments on tobacco leaf yield and quality, soil nutrient dynamics, enzyme activities, microbial biomass, and the diversity, structure, and co-occurrence networks of AOA and AOB communities. Specifically, we aimed to answer the following questions: (1) How do different tillage depths affect soil nutrient availability, biological activity over a three-year period? (2) How does tillage depth influence the diversity and community composition of AOA and AOB? (3) Does deep plowing strengthen the ecological stability and co-occurrence networks of N-cycling microbial communities? (4) What are the dominant pathways through which tillage-induced changes in soil physicochemical properties, nutrient content, and microbial communities co-regulate tobacco quality and production value? By addressing these questions and elucidating the complex pathways from tillage to crop value, this study will provide important theoretical support and practical guidance for optimizing tillage practices, improving N use efficiency, and promoting the sustainable development of agricultural ecosystems.

## 2 Materials and methods

### 2.1 Study area

The study was carried out at Xiyi Township, Longyang District, Baoshan City, Yunnan Province (99°30′E, 24°93′N), at an altitude of 1,602 m, which was a typical mountainous tobacco growing area with a subtropical monsoon climate, an average annual temperature of 16.20 °C and an average annual precipitation of 827.0 mm. The type of test soil was red soil with clay texture (17.44% sand, 26.90% silt and 55.66% clay). The basic physicochemical properties of the test soil were shown in Table 1.

**Table 1:** Basic physical and chemical properties of the test soil.

Soil layer (cm)	pH	Bulk density (g cm^−3^)	Total porosity (%)	Organic matter (g kg^−1^)	Total nitrogen (g kg^−1^)	Available phosphorus (mg kg^−1^)	Available potassium (mg kg^−1^)
0–20	8.02	1.18	52.31	32.31	2.02	41.36	310.13
20–40	8.18	1.20	50.42	28.30	1.84	18.55	181.43

### 2.2 Experimental design

**Figure 1:**
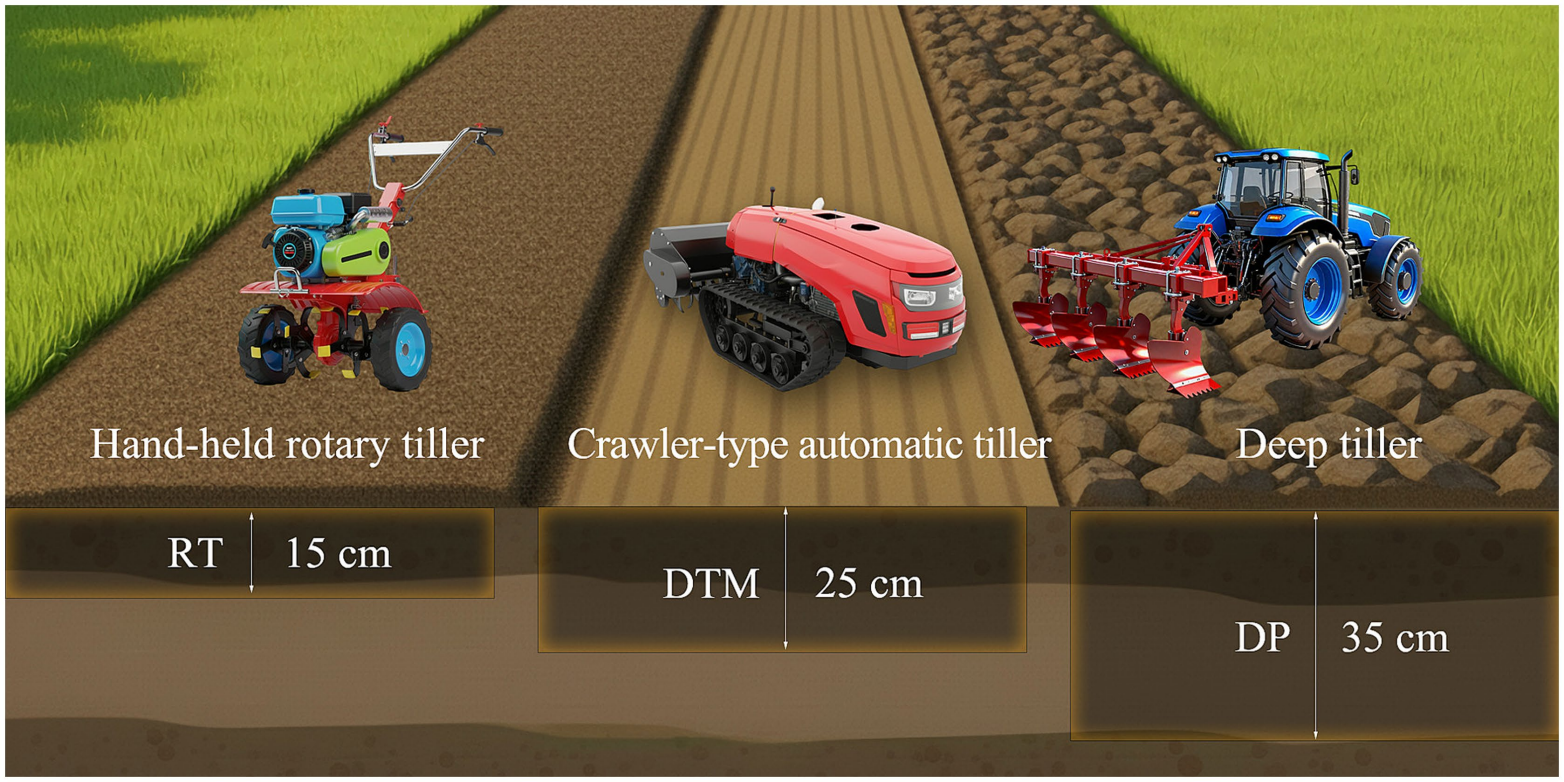
A schematic diagram of the three tillage modes implemented during the fallow season. RT (rotary tillage) was performed to a 15 cm depth with a hand-held rotary tiller (6.3 kW, model 1WG6.3); DTM (deep tillage with middle depth) was performed to a 25 cm depth with a crawler-type automatic tiller (14.7 kW, model DH1115); and DP (deep plowing) was performed to a 35 cm depth with a deep tiller (73.5 kW, model LR4A3L-23).

In this study, three tillage treatments with different tillage depths were implemented during the fallow season prior to tobacco planting from 2019 to 2021: rotary tillage (RT) with a tillage depth of 15 cm, deep tillage with middle depth (DTM) with a depth of 25 cm, and deep plowing (DP) with a depth of 35 cm (Figure 1). The experiment followed a completely randomized design with three replicates per treatment, totaling 9 plots. Each plot measured 144 m^2^ (20 m × 7.2 m). Each year, treatments were applied once before planting using machinery specific to the designated depth: a hand-held rotary tiller (6.3 kW, model 1WG6.3) for RT, a crawler-type automatic tiller (14.7 kW, model DH1115) for DTM, and a deep tiller (73.5 kW, model LR4A3L-23) for DP. Following these primary tillage operations, all plots were uniformly tilled one final time with the hand-held rotary tiller to ensure a consistent seedbed condition across all treatments. Agronomic practices were consistent for all plots. Prior to transplanting, base fertilizers were applied in strips, including 450 kg·hm^−2^ of compound fertilizer (N 7%, P_2_O_5_ 16%, K_2_O 26%), 300 kg·hm^−2^ of nitrogen-potassium fertilizer (N 16%, K_2_O 30%), and 1,200 kg·hm^−2^ of commercial organic fertilizer (N 2.77%, P_2_O_5_ 1.48%, K_2_O 1.15%). Tobacco seedlings were transplanted in early May each year, using a row spacing of 1.20 m and a plant spacing of 0.50 m. Each plot contained 240 plants, resulting in a planting density of approximately 16,667 plants hm^−2^.

### 2.3 Soil sampling

Soil samples were collected in August 2021 (during the tobacco harvest period) using a 5 cm diameter auger at two soil depths: 0–20 cm and 20–40 cm. Soil samples were taken from 10 sites arranged in an S-shape within each plot and composited into a single replicate sample after removing roots and debris. The soil samples were divided into three parts: one portion was stored at 4 °C for analysis of microbial biomass carbon (MBC), microbial biomass nitrogen (MBN), and enzyme activities; another portion (0–20 cm only) was stored at −80 °C for microbial DNA extraction and sequencing; and the remaining portion was air-dried and sieved through a 0.25 mm mesh for determination of physicochemical properties.

### 2.4 Soil properties measurements

Soil organic carbon (SOC) was determined using the potassium dichromate oxidation method (Bao, 2000). Total nitrogen (TN) was measured by the Kjeldahl digestion method (Bao, 2000). Ammonium nitrogen (NH_4_^+^-N) and nitrate nitrogen (NO_3_^−^-N) were extracted with 1 mol/L KCl and measured using a continuous flow analyzer (AA3, SEAL Analytical, Germany). Available phosphorus (AP) was determined by the molybdenum-antimony anti-colorimetric method, and available potassium (AK) was analyzed using a flame photometer (Bao, 2000). Soil pH was measured in a 1:2.5 soil-to-water suspension using a pH meter. Bulk density (BD) and soil porosity (SP) were determined by the core method (Xue et al., 2018). Soil sucrase and cellulase activities were determined by the 3,5-dinitrosalicylic acid colorimetric method (Guan et al., 1986). Urease activity was measured by the indophenol blue colorimetric method (Guan et al., 1986). MBC and MBN were determined using the chloroform fumigation-extraction method. Fresh soil samples (10 g) were fumigated with ethanol-free CHCl_3_ for 24 h at 25 °C in a vacuum desiccator. Both fumigated and non-fumigated samples were then extracted with 0.5 mol L^−1^ K_2_SO_4_ (soil-to-solution ratio of 1:4) by shaking for 30 min. The extracts were filtered and analyzed using a TOC/TN analyzer (Multi N/C 3100, Analytik Jena, Germany). MBC and MBN were calculated based on the difference in extractable C and N between fumigated and non-fumigated samples, using conversion factors of k_EC_ = 0.45 and k_EN_ = 0.54, respectively.

### 2.5 Soil DNA extraction, Illumina sequencing and bioinformatics analysis

Soil genomic DNA was extracted using the FastDNA® Spin Kit for Soil (MP Biomedicals, United States). The *amoA* genes were amplified using specific primers: Arch-amoAF (5-STAATGGTCTGGCTTAGACG-3) and Arch-amoAR (5-GCGGCCATCCATCTGTATGT-3) for AOA (Francis et al., 2005), and amoA-1F (5-GGGGTTTCTACTGGTGGT-3) and amoA-2R (5-CCCCTCKGSAAAGCCTTCTTC-3) for AOB (Rotthauwe et al., 1997). Detailed procedures for the PCR amplification process are provided in Supplementary methods. The purified amplicons were used to construct paired-end (PE) 2 × 250 bp libraries following the standard protocol for the Illumina MiSeq platform (Illumina, San Diego, United States). Sequencing was conducted on the Illumina MiSeq PE250 platform by Majorbio Bio-Pharm Technology Co., Ltd. (Shanghai, China). The bioinformatics analysis was performed using the Majorbio Cloud Platform.^1^ The raw sequencing reads were demultiplexed and quality-filtered using fastp (version 0.19.6) and merged using FLASH (version 1.2.7). Operational Taxonomic Units (OTUs) were clustered at a 97% similarity cutoff using UPARSE (version 7.1), and chimeric sequences were identified and removed. The taxonomy of each representative OTU sequence was analyzed using the RDP Classifier (Wang et al., 2007) against the FunGene functional gene database (Fish et al., 2013) with a confidence threshold of 0.7. To eliminate the effect of sequencing depth on diversity measures, the dataset was rarefied to the minimum number of sequences per sample prior to downstream analysis.

### 2.6 Determination of tobacco yield, quality, and production value

Tobacco yield was determined by harvesting, flue-curing, and weighing the leaves from 50 plants in the center of each plot. After curing, leaves were graded into superior, medium, and inferior categories to calculate the proportion of each grade. The production value was then calculated by multiplying the weight of each grade by the corresponding market price for that year (Dong et al., 2025; Hu W. et al., 2021).

### 2.7 Statistical analysis

We used two-way ANOVA to assess the effects of tillage, soil depth, and their interaction on soil physicochemical properties, enzyme activities, and microbial biomass. One-way ANOVA was used to evaluate the effect of tillage on microbial alpha diversity indices. Significant differences among treatments were identified using Tukey’s honestly significant difference (HSD) test at a significance level of *p* < 0.05. Alpha diversity indices (Shannon and Sobs) of AOA and AOB were calculated, and their taxonomic composition was visualized by relative abundance. We used Mantel tests to correlate community structure with environmental variables and Pearson correlation analysis to explore relationships between microbial diversity and soil properties. All of these analyses were conducted using the “vegan” package (Oksanen et al., 2020). To infer microbial interactions, we constructed co-occurrence networks for AOA and AOB using the “igraph” package (Csárdi and Nepusz, 2006). The networks were built from Spearman correlation matrices, retaining only strong (|*r*| > 0.6) and significant (*p* < 0.05) correlations. Key topological parameters (e.g., average degree, modularity) were calculated to characterize network complexity. Finally, we performed a Partial Least Squares Path Modeling (PLS-PM) analysis to investigate the complex causal relationships between soil properties, microbial features, and tobacco production benefits. The model included six latent variables: (1) Soil physicochemical property (pH, SP, BD); (2) Soil nutrients content (TN, AK, AP, SOC, NH_4_^+^-N); (3) Microbial community (Shannon diversity of AOA and AOB); (4) Soil microbial biomass (MBC, MBN); (5) Soil enzyme (sucrase, cellulase, urease activity); and (6) Production benefit (tobacco yield and production value). The PLS-PM was conducted using the “plspm” R package. All statistical analyses were performed using R software (version 4.4.1).

## 3 Results

### 3.1 Effects of tillage on soil nutrients and physicochemical properties

**Figure 2:**
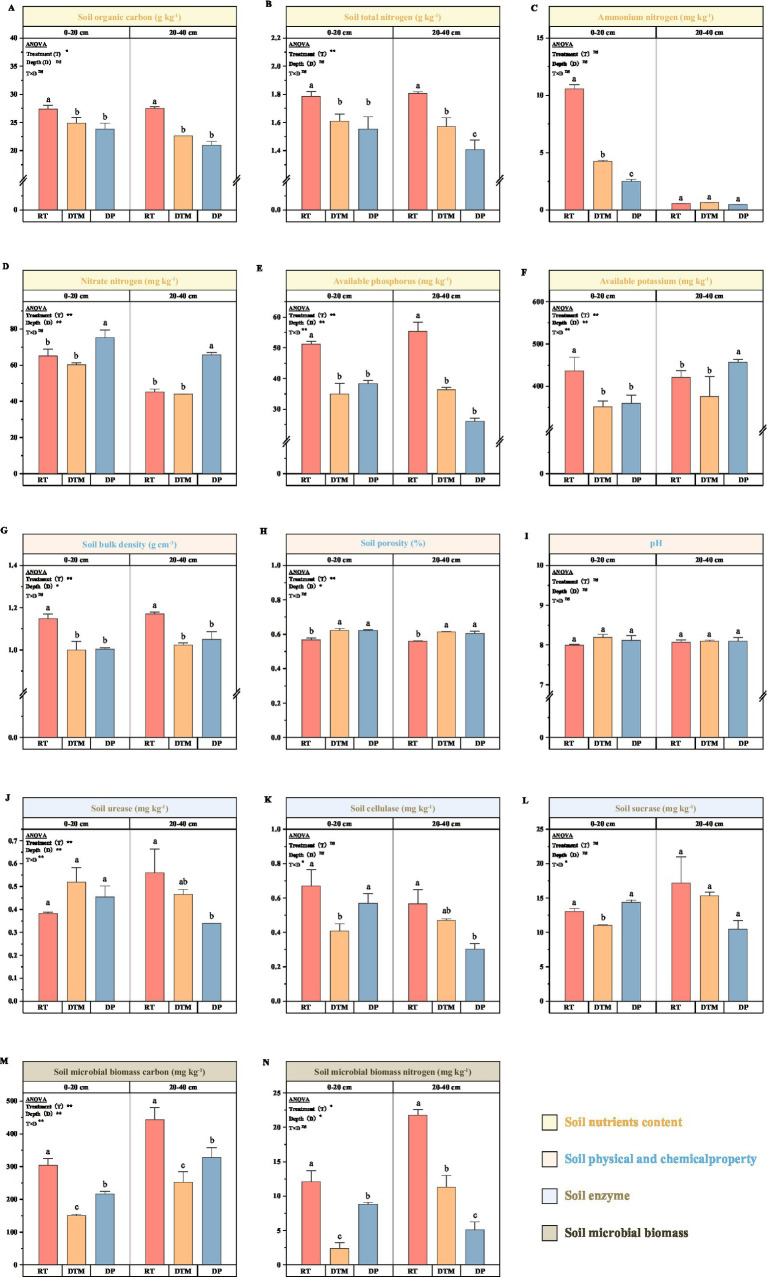
Effects of different tillage treatments on soil nutrients content **(A-F)**, physicochemical properties **(G-I)**, enzyme activities **(J-L)**, and microbial biomass **(M-N)** at two soil depths (0–20 cm and 20–40 cm). Different lowercase letters indicate significant differences among the three tillage treatments within the same soil depth (*p* < 0.05). * and ** indicate statistically significant differences at *p* < 0.05 and *p* < 0.01, respectively; ns indicates no significant difference. RT, rotary tillage; DTM, deep tillage with middle depth; DP, deep plowing.

Two-way ANOVA showed that tillage treatments significantly affected SOC, TN, NO_3_^−^-N, AP, and AK, whereas NH_4_^+^-N was not significantly affected. Soil depth had significant effects on NO_3_^−^-N, AP, and AK, and significant interactions between tillage and depth were observed for AP and AK. At the 0–20 cm depth, RT significantly increased SOC, TN, NH_4_^+^-N, AP, and AK compared to DTM and DP (Figures 2A–C,E,F). However, NO_3_^−^-N was highest under DP (Figure 2D), indicating enhanced nitrification. In the 20–40 cm layer, RT still maintained the highest SOC, TN, and AP levels (Figures 2A,B,E), while DP had the highest NO_3_^−^-N and AK (Figures 2D,F).

Tillage also significantly influenced BD and SP, both of which also varied significantly with soil depth. The interaction effect was not significant for either parameter. Compared to DTM and DP, RT significantly increased BD in both layers, reaching 1.15 g cm^−3^ at 0–20 cm and 1.17 g cm^−3^ at 20–40 cm (Figure 2G). In contrast, DTM and DP showed higher SP than RT at both depths (Figure 2H). Soil pH values were stable across treatments, ranging from 7.99–8.19 (0–20 cm) and 8.06–8.10 (20–40 cm). No significant effects of tillage, depth, or interaction were observed on pH (Figure 2I).

### 3.2 Effects of tillage on soil enzyme activities and microbial biomass

Tillage treatments had significant effects on urease activity, but not on cellulase or sucrase. Urease also varied significantly with depth, and all three enzymes showed significant interaction effects between tillage and depth. At 0–20 cm, urease activity showed no significant differences among treatments (Figure 2J). However, DTM significantly reduced cellulase and sucrase activities compared to RT and DP (Figures 2K,L). At 20–40 cm, urease and cellulase activities were significantly higher under RT than DP (*p* < 0.05), while sucrase activity showed no significant differences among treatments (Figures 2J–L).

Tillage treatments significantly affected both MBC and MBN, and soil depth had significant effects on both. A significant interaction was observed only for MBC. Across both soil layers, RT consistently produced the highest MBC values (303.82 mg kg^−1^ at 0–20 cm and 443.07 mg kg^−1^ at 20–40 cm), while DTM showed the lowest (Figure 2M). For MBN, RT also showed the highest values at both depths (Figure 2N). At 0–20 cm, MBN under DTM was the lowest, while at 20–40 cm, MBN followed the trend: RT > DTM > DP (Figure 2N).

### 3.3 Community composition and diversity of ammonia-oxidizing microorganisms under different tillage

**Figure 3:**
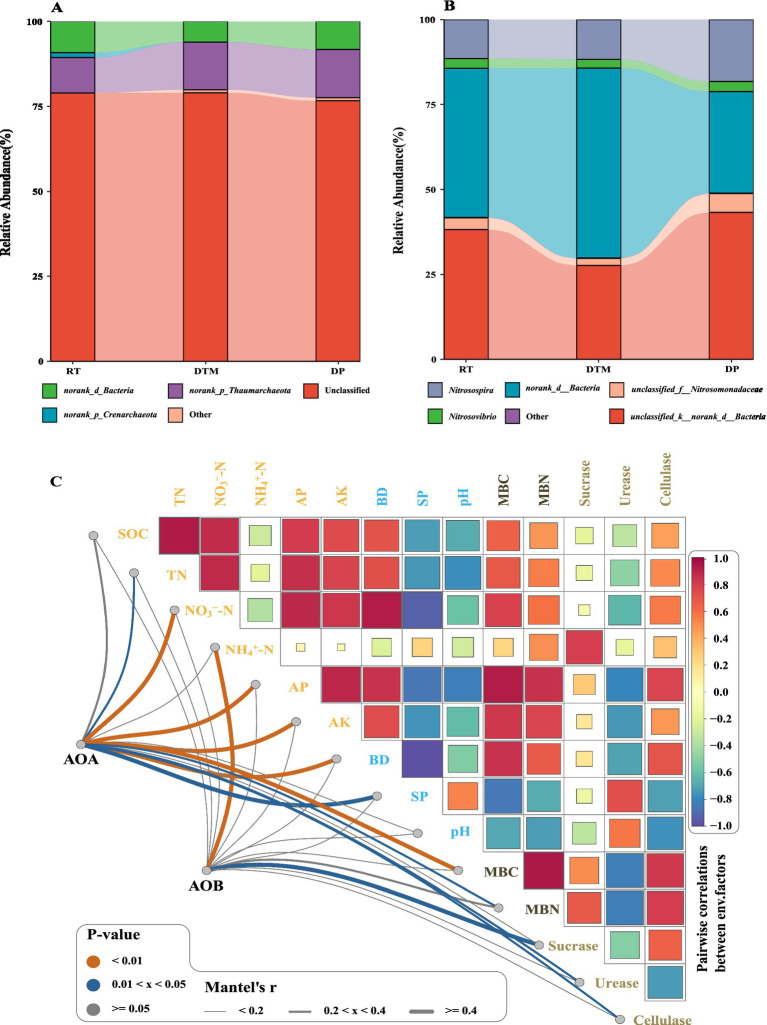
Taxonomic community composition of AOA and AOB and its correlation with soil properties. Taxonomic community composition of AOA **(A)** and AOB **(B)** at the genus level. Panel **(C)** shows the Mantel test results for correlations between soil properties and the AOA and AOB community composition at the genus level. The color gradient indicates the Mantel’s *r* statistic, with blue and red representing negative and positive correlations, respectively. The size of the squares is proportional to the strength of the correlation. RT, Rotary tillage; DTM, deep tillage with middle depth; DP, deep plowing; SOC, soil organic carbon; TN, total nitrogen; NO_3_^−^-N, nitrate nitrogen; NH_4_^+^-N, ammonium nitrogen; AP, available phosphorus; AK, available potassium; BD, bulk density; SP, soil porosity; MBC, microbial biomass carbon; MBN, microbial biomass nitrogen; AOA, ammonia-oxidizing archaea; AOB, ammonia-oxidizing bacteria.

*Norank_d_Bacteria*, *norank_p_Thaumarchacota* and *norank_p_Crenarchaeota* were the top three taxa with the highest relative abundance among the AOA (Figure 3A). Specifically, *norank_d_Bacteria* and *norank_p_Crenarchaeota* were enriched in RT, while *norank_p_Thaumarchacota* was enriched in DTM and DP. *Norank_d_Bacteria*, *Nitrosospira* and *Nitrosomonadaceae* were the top three taxa with the highest relative abundance among AOB (Figure 3B). Specifically*, norank_d_Bacteria* were enriched in DTM, while *Nitrosomonadaceae* and *Nitrosospira* were enriched in DP. Mantel analysis explained the effects of environmental factors on the community composition of AOA and AOB (Figure 3C). The community structure of AOA was influenced by NO_3_^−^-N, TN, AP, AK, BD, and SP. While NH_4_^+^-N and Sucrase mainly regulated the community structure of AOB.

**Figure 4:**
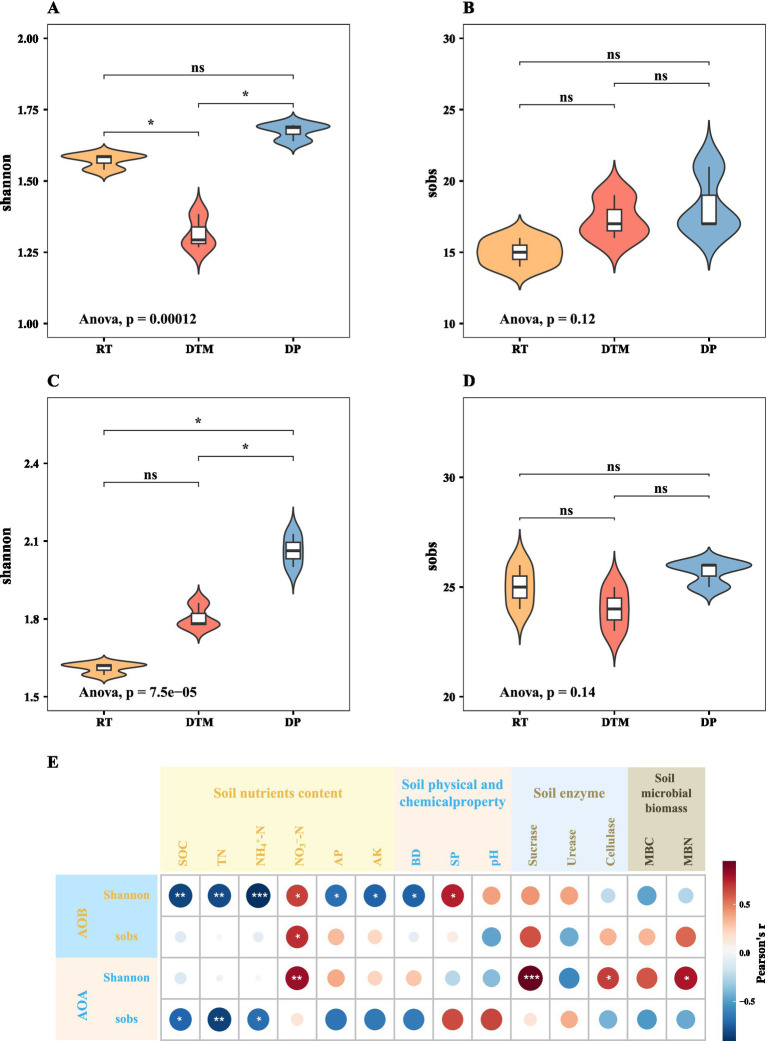
Alpha diversity of AOA and AOB communities and its correlation with soil properties. Boxplots show the Shannon and Sobs indices for AOA **(A,B)** and AOB **(C,D)** under different tillage treatments. Different lowercase letters above the boxes indicate significant differences among treatments (*p* < 0.05). Panel **(E)** displays a Pearson correlation heatmap illustrating the relationships between alpha diversity indices and soil properties. Red and blue cells represent positive and negative correlations, respectively. Significance levels are denoted by asterisks: *p < 0.05; **p < 0.01; ***p < 0.001. RT, Rotary tillage; DTM, deep tillage with middle depth; DP, deep plowing; SOC, soil organic carbon; TN, total nitrogen; NO3–-N, nitrate nitrogen; NH4+-N, ammonium nitrogen; AP, available phosphorus; AK, available potassium; BD, bulk density; SP, soil porosity; MBC, microbial biomass carbon; MBN, microbial biomass nitrogen; AOA, ammonia-oxidizing archaea; AOB, ammonia-oxidizing bacteria.

The ANOVA test for diversity revealed that the sobs indices of AOA (*p* = 0.12) and AOB (*p* = 0.14) communities were insensitive to the response of tillage treatments (Figures 4B,D). RT and DP significantly increased the Shannon index of the AOA community compared to DTM (*p* < 0.001; Figure 4A). For the AOB community, DP showed a significantly higher Shannon index than both RT and DTM (*p* < 0.001; Figure 4C). Pearson’s correlation analysis of AOA and AOB community diversity with soil properties revealed that Shannon’s index of AOB was significantly associated with soil nutrient content and soil physico-chemical properties (Figure 4E). Specifically, the Shannon index of AOB was significantly negatively correlated with SOC, TN, NH_4_^+^-N, AP, AK, and BD while it was significantly positively correlated with NO_3_^−^-N and SP. The Shannon index of the AOA community was significantly and positively correlated with NO_3_^−^-N, soil enzyme (Sucrase, Cellulase) and MBN. The sobs index of the AOB community was positively correlated with NO_3_^−^-N, whereas the sobs index of the AOA community was significantly negatively correlated with SOC, TN, NH_4_^+^-N.

### 3.4 Co-occurrence network patterns of AOA and AOB under different tillage

**Figure 5:**
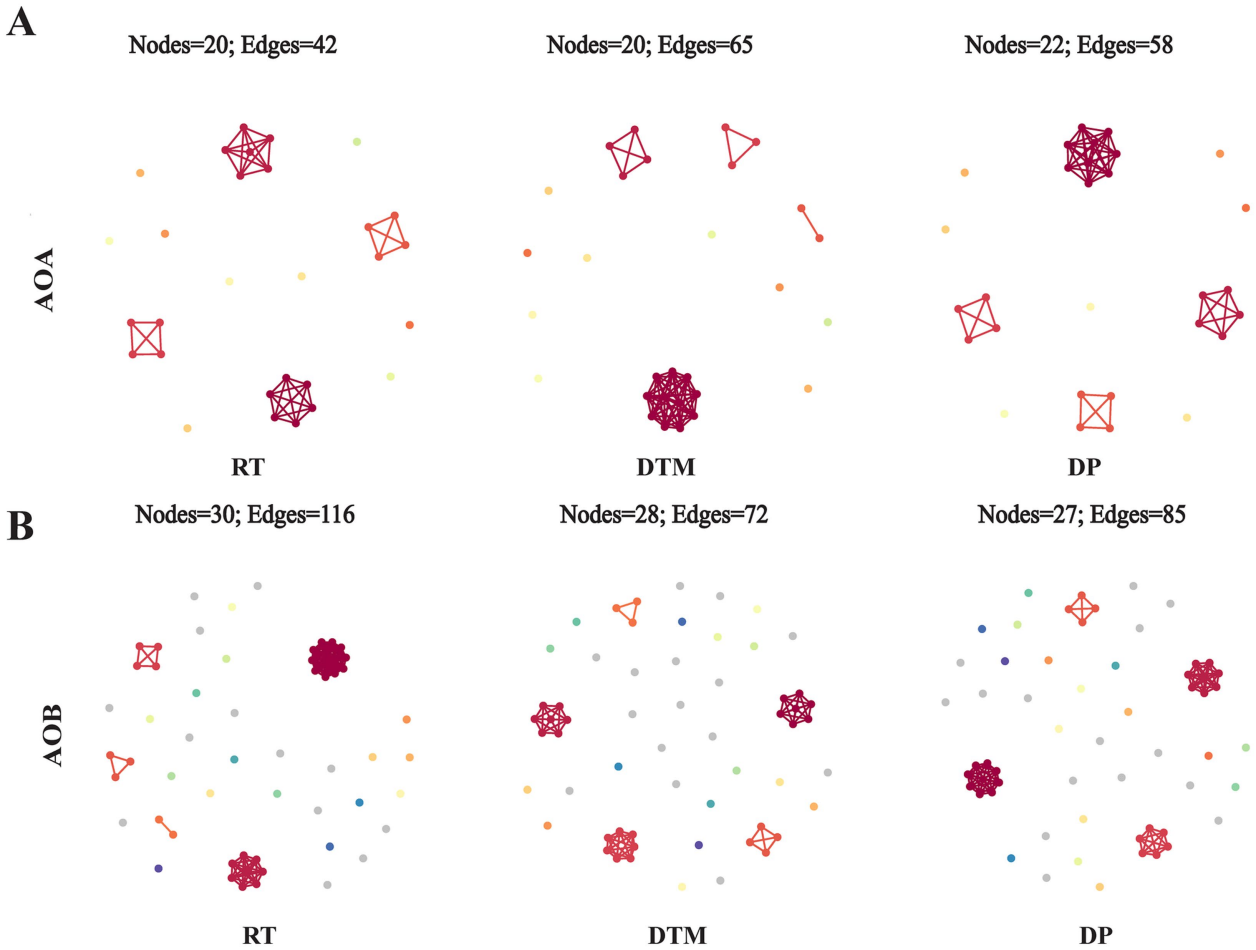
Co-occurrence networks of AOA **(A)** and AOB **(B)** communities under different tillage treatments. Key topological properties of the network are summarized in Supplementary Figure S1. AOA, ammonia-oxidizing archaea; AOB, ammonia-oxidizing bacteria. RT, rotary tillage; DTM, deep tillage with middle depth; DP, deep plowing.

Microbial co-occurrence network analysis reveals changing patterns of interactions between AOA and AOB community under different tillage treatments (Figure 5). For the co-occurring network of AOA, the tillage treatments had a limited effect on the number of nodes incorporated into the network (20–22) but improved the number of links by 54.76 and 38.06% for DTM and DP, respectively, compared to RT (Figure 5A). This implies that DTM and DP improved the interaction between AOA. However, the highest number of links in RT’s network was in the AOB community. Compared to DTM, RT and DP improved 61.11 and 18.06% of links, respectively (Figure 5B). Further analysis of the co-occurrence network properties of AOA and AOB revealed distinct patterns across tillage treatments. In the AOA network, DTM exhibited the highest average degree and edge density, but the lowest relative modularity, whereas DP had the highest relative modularity (Supplementary Figure S1A). In the AOB network, RT showed the highest average degree, centralization, and modularity, while DP again exhibited the highest relative modularity (Supplementary Figure S1B). This implied that DTM and RT improved the interaction of AOA and AOB communities, respectively, but DP improved the cohesion of modules performing potential functions in both colonies.

### 3.5 Effects of tillage on tobacco yield, value, and leaf quality

**Figure 6:**
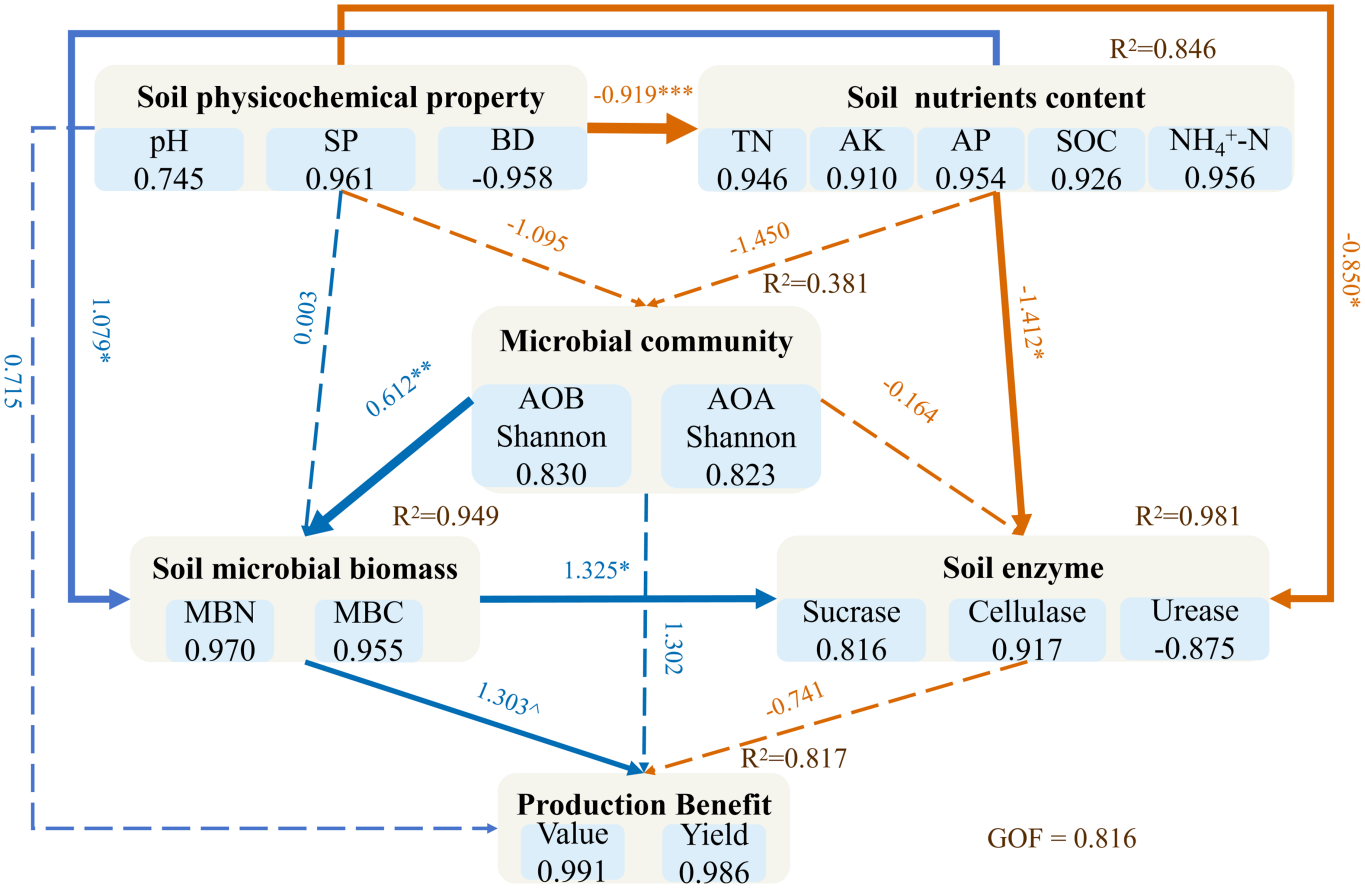
Partial least squares path modeling (PLS-PM) showing the direct and indirect pathways influencing tobacco production benefits. The model connects latent variables representing soil physicochemical property, soil nutrients content, microbial community diversity, soil microbial biomass, and soil enzyme activity to the final production benefits. Blue arrows indicate positive pathways, while orange arrows indicate negative pathways. Numbers on the arrows are the standardized path coefficients. Asterisks denote the level of significance: ^*p* < 0.10, *p < 0.05, **p < 0.01, ***p < 0.001. The R^2^ values next to the endogenous variables indicate the proportion of variance explained by the model. The overall Goodness-of-it (GOF) for the model is shown at the bottom. SP, soil porosity; BD, bulk density; TN, total nitrogen; AK, available potassium; AP, available phosphorus; SOC, soil organic carbon; NH_4_^+^-N, ammonium nitrogen; AOA, ammonia-oxidizing archaea; AOB, ammonia-oxidizing bacteria; MBC, microbial biomass carbon; MBN, microbial biomass nitrogen.

Tillage treatments had no statistically significant effect on the overall tobacco yield or production value, although the highest numerical values for both were observed under the deep plowing (DP) treatment (2,825 kg hm^−2^ and 88,959 CNY hm^−2^, respectively) (Table 2). However, tillage significantly impacted the quality distribution of tobacco leaves. The proportion of superior-grade leaves was significantly higher under DP (77.15%) compared to rotary tillage (RT) (68.82%). Conversely, there were no significant differences among treatments for the proportions of medium-grade or inferior-grade leaves. The PLS-PM analysis indicated that soil physicochemical properties, nutrient content, and microbial characteristics collectively explained 81.7% of the variance in production benefits (*R*^2^ = 0.817) (Figure 6). Specifically, physicochemical properties, including soil pH, porosity, and bulk density, affected production benefits indirectly via soil nutrients and soil microbial biomass. Soil nutrients and the diversity of AOA and AOB communities exerted a significant positive direct effect on soil microbial biomass. In turn, soil microbial biomass not only directly and significantly promoted soil enzyme activity (standardized path coefficient = 1.325, *p* < 0.05) but also demonstrated the strongest positive direct influence on production benefits (standardized path coefficient = 1.303, *p* < 0.10).

**Table 2:** Effects of tillage treatments on yield, production value, and leaf grade distribution of cured tobacco.

Treatment	Yield (kg hm^−2^)	Production value (CNY hm^−2^)	Proportion of superior tobacco leaves (%)	Proportion of medium-grade tobacco leaves (%)	Proportion of inferior tobacco leaves (%)
RT	2585.00 ± 78.07 a	80445.20 ± 2445.39 a	68.82 ± 2.15 b	19.72 ± 1.05 a	11.46 ± 3.23 a
DTM	2505.00 ± 17.32 a	80841.40 ± 948.31 a	72.99 ± 0.29 ab	19.08 ± 0.13 a	7.93 ± 0.42 a
DP	2825.00 ± 122.56 a	88959.25 ± 3822.42 a	77.15 ± 0.58 a	18.44 ± 0.33 a	4.41 ± 0.41 a

Different lowercase letters indicate significant differences among the three tillage treatments within the same soil depth (*p* < 0.05). RT, rotary tillage; DTM, deep tillage with middle depth; DP, deep plowing.

## 4 Discussion

### 4.1 Tillage depth significantly affects soil nutrient stratification and physical properties

Our results showed that RT significantly increased SOC, TN, AP, and AK in the 0–20 cm soil layer compared to DTM and DP. The increase in SOC is primarily attributed to the reduced soil disturbance under RT, which minimizes soil aeration and slows the mineralization of organic matter, thereby facilitating the retention of crop residues in the surface layer (Lv et al., 2023; Shen et al., 2024). In contrast, the accumulation of TN, AP, and AK is largely driven by the shallow incorporation of basal fertilizers. Since rotary tillage restricts soil mixing to the top 15 cm, the applied nitrogen, phosphorus, and potassium remain concentrated in the surface soil. In the 20–40 cm layer, RT still maintained higher SOC, TN, and AP levels compared to DTM and DP, which contrasts with studies suggesting that deep tillage enhanced subsoil fertility by facilitating nutrient redistribution (Feng et al., 2020; Li J. et al., 2024; Qiao et al., 2025). This inconsistency may be explained by differences in experimental duration, residue input, and rooting depth of crops. While long-term deep tillage combined with deep-rooted crops and high biomass return generally improves subsoil nutrient availability, our relatively short (3-year) tobacco field experiment, characterized by low residue input and shallow root systems, likely limited nutrient translocation to deeper layers. Regarding potassium, RT increased AK in the 0–20 cm layer, whereas DP significantly elevated AK in the 20–40 cm layer, reflecting the enhanced mixing and downward movement of potassium under deep tillage as reported previously (Mallarino and Borges, 2006).

RT significantly increased BD in both the 0–20 cm and 20–40 cm layers, while significantly reducing SP, indicating that RT resulted in a less optimal physical environment with reduced air and water movement. The resulting higher bulk density and lower porosity reduced oxygen diffusion in the soil, suppressing nitrification, as further indicated by the lower NO_3_^−^-N concentrations and leading to greater accumulation of NH_4_^+^-N in the 0–20 cm surface layer (Pengthamkeerati et al., 2006). In contrast, DP significantly decreased bulk density and increased porosity in both soil layers, improving soil aeration and moisture conditions (Yang et al., 2021). This enhanced aeration likely accelerated the oxidative decomposition of soil organic carbon, contributing to the significantly lower SOC levels observed under DP. This improved soil physical environment favored nitrifying microbes, promoting the conversion of NH_4_^+^-N to NO_3_^−^-N (Pengthamkeerati et al., 2006), which resulted in significantly higher NO_3_^−^-N concentrations in both the 0–20 cm and 20–40 cm layers compared to RT and DTM.

### 4.2 Soil biological activity is modulated by tillage depth and soil environment

Soil enzyme activities and microbial biomass are key indicators of biological quality and nutrient cycling (Borny et al., 2024; Neemisha and Sharma, 2022; Nikitin et al., 2022; Yu et al., 2018). In our study, RT generally enhanced these properties compared to DTM and DP. At the 0–20 cm layer, urease activity did not differ significantly among treatments, likely reflecting the buffering capacity of established microbial communities and regulation by other factors such as pH (Fisher et al., 2017). However, cellulase and sucrase activities were significantly reduced under DTM compared to RT and DP, which may be attributed to the decreased availability of organic substrates, as indicated by the lower SOC in the DTM treatment. This limitation in substrate availability likely constrained enzyme activity. In the 20–40 cm subsoil, urease and cellulase activities were significantly higher under RT than DP, suggesting that RT preserved favorable conditions for microbial activity at depth, possibly by minimizing physical disturbance and maintaining substrate availability (Shen et al., 2024). DP, on the other hand, may have disrupted aggregate structures, impairing microbial habitat quality (Basic et al., 2004; Dai et al., 2021).

RT also produced the highest MBC and MBN across both depths, consistent with findings that reduced tillage promotes microbial biomass by preserving structure and reducing disturbance (Chen et al., 2019; Dhaliwal et al., 2024; Yu et al., 2025). The lowest MBC at both 0–20 cm and 20–40 cm, along with the lowest MBN at 0–20 cm under DTM, may indicate a “dual nutrient limitation” mechanism (Demoling et al., 2007). RT maintained the highest microbial biomass primarily due to the enrichment of organic substrates (SOC) in the surface layer. In contrast, DTM diluted these organic substrates through mixing, limiting heterotrophic growth. Furthermore, unlike DP which compensated for carbon loss with significantly enhanced nitrate (NO_3_^−^-N) availability, DTM failed to generate comparable inorganic nitrogen enrichment. Consequently, the soil environment under DTM lacked both the concentrated labile carbon of RT and the high nitrate levels of DP, severely restricting overall microbial proliferation (Manzoni et al., 2012).

### 4.3 Community shifts in AOA and AOB are governed by tillage-induced environmental gradients

AOA and AOB play essential roles in soil N cycling, and their community composition is highly responsive to soil management practices such as tillage (Haiming et al., 2022; Li et al., 2015; Liu et al., 2018). In our study, different tillage treatments selectively enriched dominant ammonia-oxidizing microbial groups, reflecting the environmental selection imposed by tillage practices that shape microbial community composition (Khan et al., 2023). Mantel analysis further demonstrated that AOA communities were closely associated with NO₃^−^-N, TN, AP, AK, BD, and SP, while AOB communities were mainly influenced by NH₄^+^-N and sucrase activity. This indicates a functional niche differentiation between AOA and AOB, with AOA more influenced by soil nutrient status and physical conditions, whereas AOB respond mainly to immediate NH₄^+^ availability and labile carbon sources (Carey et al., 2016; Sterngren et al., 2015).

Although the sobs index of both AOA and AOB showed no significant response to tillage, the Shannon diversity index, which accounts for both richness and evenness, was significantly affected. Specifically, RT and DP significantly enhanced AOA diversity compared to DTM, while AOB diversity was highest under DP. These results suggest that tillage intensity influences microbial diversity patterns, with RT and DP promoting greater diversity likely by maintaining or improving habitat heterogeneity and resource availability (Yu et al., 2025; Zhang et al., 2022). This reduction in diversity under DTM can also be explained by the ‘dual nutrient limitation’ mechanism described above (Demoling et al., 2007). While RT supported diverse heterotrophic populations through abundant surface carbon, and DP fostered specific nitrifying communities via high nitrate availability, DTM offered neither resource advantage. This scarcity of both organic substrates and inorganic nitrogen likely created a stressful, homogeneous environment that intensified competitive exclusion among microbial taxa, thereby inhibiting the coexistence of diverse species and resulting in the lowest community diversity. This highlights the importance of considering both the nature and intensity of soil management practices on the complex responses of microbial communities (Chernov and Semenov, 2021). Correlation analysis revealed that AOA and AOB diversity were distinctly influenced by different soil nutrients, enzyme activities, and physical properties, suggesting that they occupy complementary niches that promote coexistence and stabilize soil N cycling under varying conditions.

It is important to note that while soil physicochemical properties were assessed at two depths (0–20 cm and 20–40 cm), the microbial community analysis (including DNA extraction and sequencing of AOA and AOB) in this study was restricted to the 0–20 cm soil layer. Therefore, the observed shifts in AOA and AOB diversity and community structure directly reflect the response of the topsoil community to tillage disturbances. Although the improved soil porosity and significant nitrate accumulation observed in the 20–40 cm subsoil under DP suggest that deep plowing may also favorably alter the subsoil microbial niche, direct molecular evidence for these deep-layer changes was not obtained in this study. Future research involving deep-profile sequencing is necessary to fully elucidate the vertical spatial dynamics of nitrogen-cycling microorganisms under different tillage depths.

### 4.4 Microbial co-occurrence networks reveal differential structural stability under tillage treatments

Microbial co-occurrence networks offer valuable insights into ecological interactions, cooperation, and competition among microbial taxa in response to environmental changes (Gao et al., 2022; Hu X. et al., 2021; Li et al., 2021). In this study, different tillage practices altered the complexity and structural characteristics of both AOA and AOB networks, reflecting their distinct responses to soil disturbance. The number of AOA nodes remained stable across treatments, indicating a relatively consistent core community structure. However, both DTM and DP enhanced potential interactions among AOA taxa, suggesting that moderate disturbance and nutrient redistribution may promote network connectivity by creating more heterogeneous niches and increasing metabolic interdependence (Cornell et al., 2023; Song et al., 2023). The DTM network exhibited high connectivity but low modularity, implying greater ecological redundancy and cooperative potential among AOA taxa, whereas the high modularity observed under DP may result from microsites with distinct physicochemical conditions, driving niche differentiation and modular assembly (Guseva et al., 2022). In contrast, RT generated the most complex and centralized AOB network, indicating that low disturbance may maintain stable substrate supply and habitat structure, thereby facilitating cooperative interactions among functional groups (Hu et al., 2020). DP also showed relatively high modularity in the AOB network, possibly linked to improved oxygen availability and enhanced nitrification activity in deeper soil layers (Hu et al., 2024). Overall, these findings indicate that tillage practices not only influence the strength of interactions within AOA and AOB communities but also shape their organizational patterns, potentially affecting the stability and functional performance of soil N cycling.

### 4.5 Oil properties and microbial pathways co-regulate tobacco yield and quality

Although tillage treatments did not produce statistically significant differences in total tobacco yield, DP demonstrated a clear advantage by yielding a significantly higher proportion of superior-grade leaves. This finding aligns with previous research suggesting that intensive tillage primarily benefits crop production by improving the soil’s physical environment and ecological functions (Huang et al., 2023). Practices like DP improve the soil’s physical structure (e.g., increasing porosity, reducing bulk density), which is a critical determinant of habitat for soil microorganisms (Six et al., 2004). These physical improvements indirectly enhance production benefits by positively influencing soil nutrient availability and the microbial community. PLS-PM shows that soil microbial biomass (MBC and MBN) was identified as the strongest direct positive driver of production benefits. As a key indicator of soil health (Wardle, 1992), microbial biomass directly boosted soil enzyme activity, accelerating nutrient turnover for plant uptake (Nannipieri et al., 2017) and promoting high-quality crop growth. However, it is important to note that while RT maintained the highest total microbial biomass, it did not yield the highest proportion of superior-grade leaves. This discrepancy implies that crop quality may not be driven solely by the total magnitude of biomass (MBC/MBN), but is likely the result of the synergistic interaction between microbial activity and an optimized soil environment (Bhandari et al., 2025). Unlike RT, DP improved soil aeration and porosity, which appeared to enhance the metabolic efficiency of the microbial community and favored specific functional groups (such as AOB) essential for nitrogen transformation. Thus, the coordination between microbial biomass and favorable physical conditions under DP may be more critical for determining leaf quality than the sheer biomass quantity observed under RT. Notably, while this accelerated nutrient turnover is crucial for current crop quality, it may also imply a potential risk of soil organic matter depletion over the long term. Therefore, integrating organic amendments with deep plowing strategies is recommended to compensate for carbon consumption and ensure sustainable agricultural production. This underscores the importance of cultivating a healthy soil ecosystem for high-value, sustainable production (Doran and Zeiss, 2000).

## 5 Conclusion

This study demonstrated that tillage depth strongly regulates soil physical structure, nutrient stratification, and N-cycling microbial communities in a subtropical red soil region. RT enhanced soil organic carbon, total nitrogen, available phosphorus, and microbial biomass carbon and nitrogen, but caused higher bulk density and lower porosity. In contrast, DP reduced nutrient and microbial biomass levels but improved soil porosity and enhanced the diversity of AOB, indicating improved aeration and nitrification activity. Although total yield and production value did not differ significantly among treatments, DP increased the proportion of superior-grade leaves, suggesting improved crop quality rather than quantity. Path analysis further identified soil microbial biomass as the key mediator linking tillage-induced changes in soil physicochemical properties to production benefits. These findings highlight that different tillage depths achieve agronomic outcomes through distinct mechanisms: RT favors nutrient enrichment and biological activity, while DP optimizes soil structure and N transformation to improve leaf quality. Given the potential for soil organic matter depletion under deep plowing, integrating organic amendments with DP is recommended to sustain long-term soil fertility. Overall, adopting appropriate tillage depth according to local soil conditions can enhance soil ecological functions and support sustainable agricultural management in subtropical red soil regions.

## Data Availability

The original contributions presented in the study are included in the article/Supplementary material, further inquiries can be directed to the corresponding authors.

## Footnotes

1
https://cloud.majorbio.com
