# Supplementary material for: Deep plowing improves soil physical structure and alters nitrogen-cycling microbial communities in a subtropical red soil region
Source: Front Microbiol. 2026 Jan 12;16:1734649. doi: 10.3389/fmicb.2025.1734649 (PMC12833330; doi:10.3389/fmicb.2025.1734649)
Supplement: Supplementary file 1 [file Data_Sheet_1.docx]

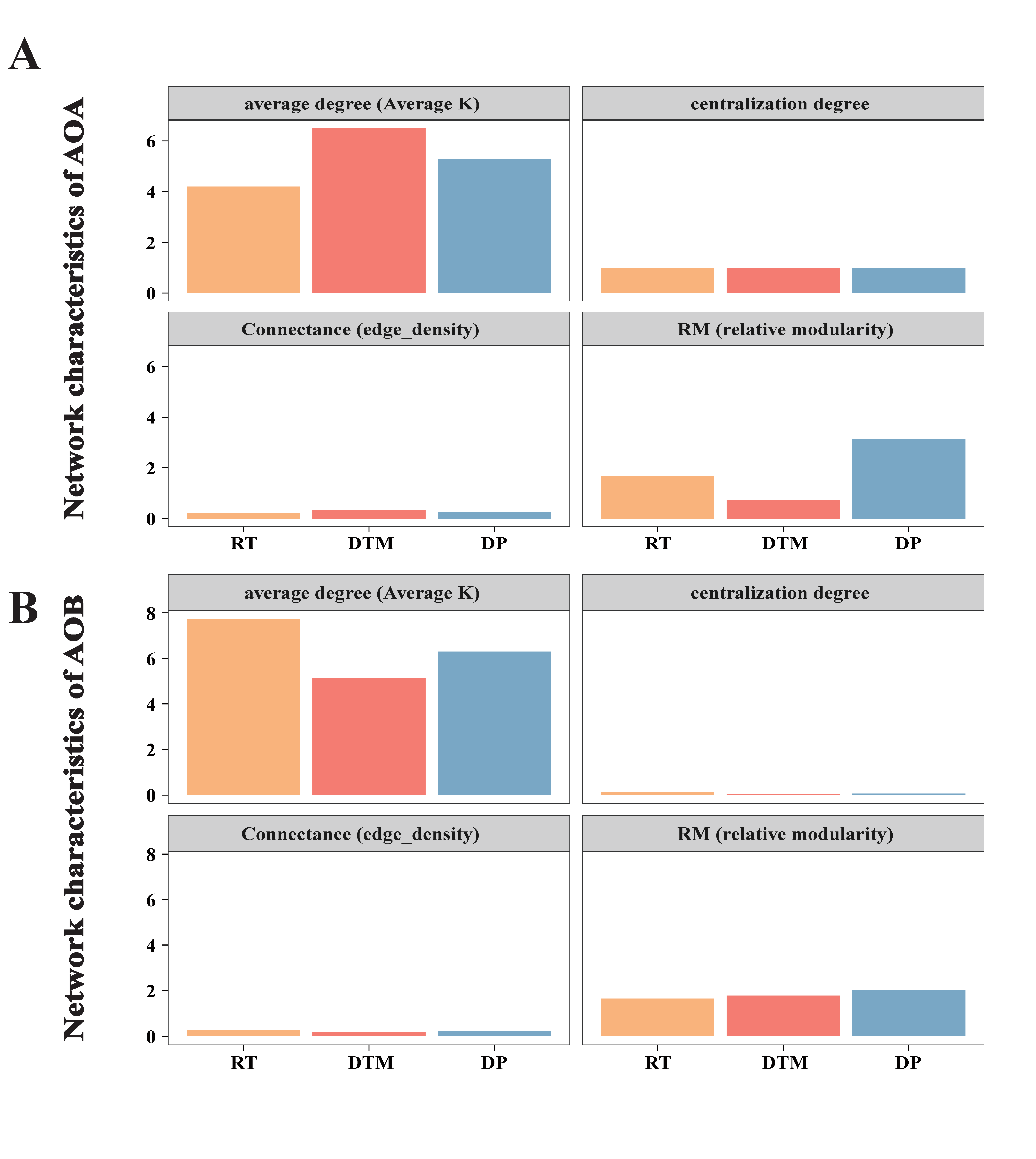


**Fig. S1** Key topological properties of the network in ammonia-oxidizing archaea (A) and ammonia-oxidizing bacteria (B). RT, rotary tillage; DTM, deep tillage with middle depth; DP, deep plowing.

Supplementary Methods

The PCR reactions were performed in a 20 μL mixture containing 4 μL of 5× FastPfu Buffer, 2 μL of 2.5 mM dNTPs, 0.8 μL of each primer (5 μM), 0.4 μL of FastPfu Polymerase, and 10 ng of DNA template. Each sample was amplified in triplicate. The thermal cycling conditions were as follows: initial denaturation at 95 °C for 5 min; followed by 40 cycles for AOA and 35 cycles for AOB of denaturation at 95 °C for 30 s, annealing at 55 °C for 30 s, and extension at 72 °C for 45 s; with a final extension at 72 °C for 10 min. The PCR products were extracted from a 2% agarose gel and purified using the AxyPrep DNA Gel Extraction Kit (Axygen Biosciences, USA) according to the manufacturer's instructions.
